# Effects of Cerebellar Non-Invasive Stimulation on Neurorehabilitation in Stroke Patients: An Updated Systematic Review

**DOI:** 10.3390/biomedicines12061348

**Published:** 2024-06-18

**Authors:** Qi Liu, Yang Liu, Yumei Zhang

**Affiliations:** 1 Department of Neurology, Beijing Tiantan Hospital, Capital Medical University, Beijing 100070, China; 2 Department of Rehabilitation, Beijing Tiantan Hospital, Capital Medical University, Beijing 100070, China; 3 China National Clinical Research Center for Neurological Diseases, Beijing Tiantan Hospital, Capital Medical University, Beijing 100070, China

**Keywords:** cerebellar stimulation, stroke, rehabilitation, motor function, dysphagia, aphasia

## Abstract

The cerebellum is emerging as a promising target for noninvasive brain stimulation (NIBS). A systematic review was conducted to evaluate the effects of cerebellar NIBS on both motor and other symptoms in stroke rehabilitation, its impact on functional ability, and potential side effects (PROSPERO number: CRD42022365697). A systematic electronic database search was performed by using PubMed Central (PMC), EMBASE, and Web of Science, with a cutoff date of November 2023. Data extracted included study details, NIBS methodology, outcome measures, and results. The risk of bias in eligible studies was also assessed. Twenty-two clinical studies involving 1016 participants were finally included, with a focus on outcomes related to post-stroke motor recovery (gait and balance, muscle spasticity, and upper limb dexterity) and other functions (dysphagia and aphasia). Positive effects were observed, especially on motor functions like gait and balance. Some efficiency was also observed in dysphagia rehabilitation. However, findings on language recovery were preliminary and inconsistent. A slight improvement in functional ability was noted, with no serious adverse effects reported. Further studies are needed to explore the effects of cerebellar NIBS on post-stroke non-motor deficits and to understand how cerebellar engagement can facilitate more precise treatment strategies for stroke rehabilitation.

## 1. Introduction

Stroke is still the most common cause of death and disability, although its mortality is decreasing [1]. Patients suffering stroke often experience varying degrees of motor and other function impairments. Hemiplegia, stiffness, and postural instability are the cardinal motor signs of stroke. Other symptoms such as aphasia, cognitive impairment, and mental health problems are also very common in patients with stroke [2]. These issues may also have a greater impact on patients’ functional ability [3].

To this day, there is larger demand for rehabilitation treatments due to the rising number of stroke survivors, and the importance of rehabilitation has been recognized in treating the sequelae of stroke [4]. Various rehabilitation strategies have been used, including traditional therapies like physiotherapy and speech therapy, as well as robot-assisted training [5] and virtual reality training [6]. However, the effects of the above stroke rehabilitation strategies are still limited.

Insights from studying neural circuits that control movement, speech, and behavior after stroke have led to innovative rehabilitation approaches [7]. One promising novel strategy is the combination of behavioral training with noninvasive brain stimulation (NIBS), including transcranial magnetic stimulation (TMS), transcranial direct current stimulation (tDCS), transcranial alternating current stimulation (tACS), transcranial focused ultrasound stimulation (tFUS), transcranial unfocused ultrasound stimulation (tUUS), and transcutaneous vagus nerve stimulation (tVNS), etc. [7,8,9]. Several meta-analyses have indicated the positive effects of NIBS on functional recovery in stroke patients [10,11,12,13,14,15,16]. Orrù et al. and Li et al. found NIBS to be effective in improving upper extremity motor function and activities of daily life after stroke, with the most significant intervention effect observed with anodal-tDCS [10,11]. O’Brien et al. further demonstrated that NIBS is linked to improvements in fine motor performance in chronic stroke patients, supporting its role in enhancing motor learning [12]. Additionally, Chen et al.’s review highlighted that stimulation of the primary motor cortex and/or primary somatosensory cortex significantly enhanced post-stroke sensory dysfunction [13]. And, evidence from other meta-analyses has also demonstrated the beneficial effects of NIBS in stroke patients with aphasia [14], cognitive impairment [15], and emotional disorders such as depression [16].

As of now, the primary targets of NIBS continue to focus on supratentorial regions, such as the primary motor cortex (M1) for enhancing motor function and the Broca area for speech improvement [14,17]. However, some studies have not yielded satisfactory results [17]. Over the past thirty years, the cerebellum has been confirmed to play a role in regulating motor and non-motor function [18,19]. Cortico-cerebellar circuits are associated with motor learning and feedforward control, as well as cognitive regulation [20,21]. Because the cerebellum provides unique plasticity mechanisms and has vast connections to cortical areas, it has been served as a target of NIBS [22,23]. Cerebellar NIBS can induce changes in cerebellar excitability and modulate brain function via the cerebellar-thalamo-cortical loop, as well as support the restoration of lost abilities through intrinsic learning processes [2,24]. In a recent meta-analysis study, the comparative effectiveness of stimulation targeting the motor cortex and cerebellum revealed similar outcomes in motor function recovery, suggesting that the cerebellum holds potential as a target for NIBS [25].

Stroke results in disruptions in brain networks, notably affecting the cortico-cerebellar system [26]. Although studies have demonstrated the efficacy of cerebellar stimulation in stroke rehabilitation, they are limited by small sample sizes and the use of single outcome measures [2]. As of now, cerebellar NIBS has not been integrated into standardized stroke rehabilitation protocols. Although two recent systematic reviews have addressed the application of cerebellar NIBS, these have focused either solely on cerebellar tDCS or on exploring various stimulation types in neurorehabilitation [27,28]. These reviews have not thoroughly examined the impact of cerebellar NIBS on a range of post-stroke neurologic deficits. Moreover, studies post-2022 have highlighted the efficacy of cerebellar NIBS in treating dysphagia [29,30,31,32], fine motor dysfunction [33,34], aphasia [35], and cognitive impairment [36] after stroke, showcasing the potential of cerebellar stimulation beyond treating dyskinesia. Thus, an updated systematic review that comprehensively evaluates the diverse stimulation parameters of NIBS for varying post-stroke sequelae is essential to promote its broader application in stroke rehabilitation. In this review, we aim to explore the efficacy of cerebellar NIBS as a therapeutic intervention for enhancing motor functions and other function deficits in stroke patients. The safety and tolerability of this intervention will also be assessed.

## 2. Materials and Methods

This systematic review followed the *Preferred Reporting Items for Systematic Reviews and Meta-Analyses* (PRISMA) statements [37].

### 2.1. Search Strategy

The study protocol was registered in the International Prospective Register of Systematic Reviews (PROSPERO) with the registration number CRD42022365697. A comprehensive search was conducted in databases including PubMed, EMBASE, and Web of Science to identify relevant studies until 1 November 2023. The search terms used included Medical Subject Headings (MeSH) and Emtree headings combined with three keywords: stroke, stimulation, and cerebellum (Table S1).

### 2.2. Study Selection

We followed the PICOS criteria to formulate the main question for this systematic review. Our inclusion criteria were: P (population): patients with stroke; I (interventions): cerebellar NIBS; C (comparators): sham cerebellar stimulation or no use of the device; O (outcomes): motor outcome and/or other outcome; and S (study designs): randomized controlled trials or other types of clinical studies. Our analysis excluded conference abstracts, review articles, case reports with sample sizes of less than five, letters, animal studies, and in vitro studies. Studies with duplicate or overlapping data were also excluded. The study selection process was conducted independently by two reviewers and any disagreements were resolved through discussion.

### 2.3. Data Extraction

After reading the full text, two reviewers independently extracted relevant data and cross-checked it. The extracted content mainly included the first author, year of publication, population sample size, the demographic characteristic of the included articles (lesion site and disease duration), the methodology of each research study (type of stimulation, parameters, location, and number of training sessions), measurements of outcomes, and the results.

### 2.4. Assessing Risk of Bias in Included Studies

Two independent researchers assessed the risk of bias and quality of each included article using the Cochrane Risk of Bias Tool [38]. The evaluation of bias included seven categories that are commonly evaluated in parallel group trials: random sequence generation, allocation concealment, blinding of participants and personnel, blinding of outcome assessments, incomplete outcome data, selective reporting, and other potential sources of bias (e.g., eligibility criteria, baseline similarity, consistency of co-interventions, etc.).

## 3. Results

### 3.1. Studies Included and Excluded

We conducted a comprehensive literature search and identified a total of 857 articles (PubMed, 165; EMBASE, 135; Web of Science, 557). After removing duplicates, we narrowed down the search to 667 articles. Based on title and abstract screening, 621 publications were excluded.

**Figure 1:**
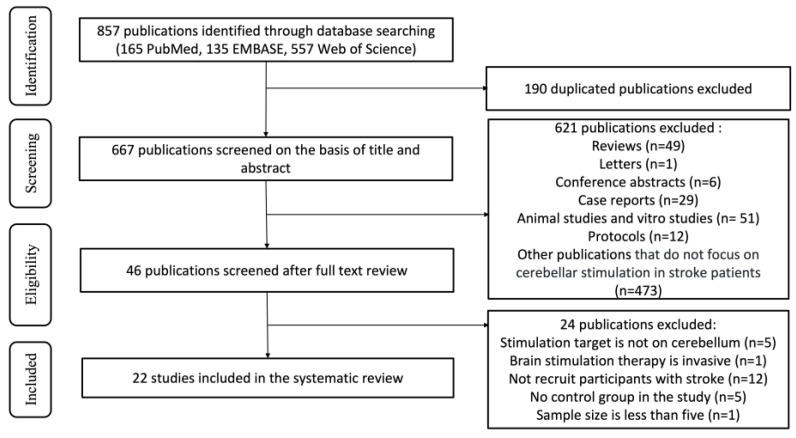
Flow diagram of the study.

Among these articles, 148 were excluded due to their type, and another 473 were excluded because their topics were irrelevant, such as having unrelated intervention therapies (for example, acupuncture, and conventional physiotherapy) and unrelated outcomes (like changes in brain area activity and perfusion). The remaining 46 full-text articles were then reviewed in their entirety, and ultimately 22 clinical studies specifically concerning the use of cerebellar NIBS in stroke rehabilitation were assessed for eligibility for inclusion in this systematic review (Figure 1).

### 3.2. Bias Analysis

**Figure 2:**
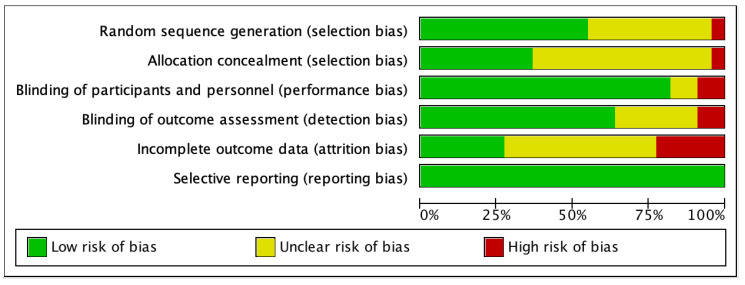
Risk of bias graph. Review authors’ assessments of each risk of bias item presented as percentages across all included studies.

**Figure 3:**
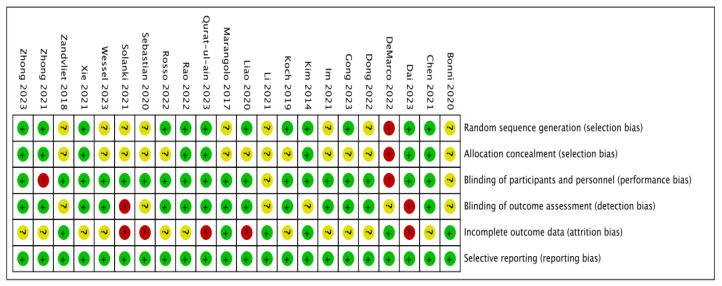
Risk of bias summary. Review authors’ judgments on each risk of bias item for every included study [29,30,31,32,33,34,35,36,39,40,41,42,43,44,45,46,47,48,49,50,51,52].

Several biases were detected that may affect the analysis of the results (Figure 2 and Figure 3, Table S2). The review included twenty-one randomized controlled trials (RCTs) and one pilot study [35]. Among the RCTs, fifteen were parallel-design [29,30,31,32,34,36,39,40,41,42,43,44,45,46,47], while six were crossover-design [33,48,49,50,51,52]. Except for the pilot study, all RCT experiments utilized random assignment and allocation concealment. Fifteen RCTs blinded both participants and outcome assessors [29,30,32,33,34,36,39,40,41,43,44,46,47,48,51], while two RCTs, focusing on aphagia, were single-blinded examiner studies [27,33]. In two studies, blinding was applied to participants, but the blinding status of the outcome assessors was not mentioned in the articles [49,52]. The blinding status was not specified in the remaining two studies [42,50]. The pilot study focusing on aphasia did not employ blinding [35]. In terms of attrition bias, six studies reported no dropouts [35,39,42,48,49,50], eleven studies had dropouts below 10% [29,30,32,33,34,40,41,44,45,46,47], and five studies had dropouts exceeding 10% [31,36,43,51,52].

### 3.3. Study Characteristics

A total of 1016 subjects were included in the analysis. The number of participants randomized in each individual trial ranged from 16 to 143. With the exception of three studies (two focusing on gait and one on dysarthria) that included subjects with infratentorial infarcts [31,39,50]; all other studies included patients with supratentorial infarcts [29,30,32,33,34,35,36,39,40,41,42,43,44,45,46,47,48,49,51,52]. All post-stroke aphasia (PSA) patients included in the current review were right-handed patients with left hemispheric infarcts [35,48,51]. Eight studies were conducted in the acute to early subacute stroke phase (0–3 months) [29,30,31,32,39,41,43,45], thirteen studies were conducted in the chronic phase (>3 months) [33,34,35,40,42,44,46,47,48,49,50,51,52], and one study did not specify the timing of cerebellar NIBS treatment [36]. In total, fourteen studies examined the effects of cerebellar NIBS on motor recovery following stroke, focusing on gait and balance, muscle spasticity, and hand function. Eight studies investigated other functions, with five studies addressing dysphagia [29,30,31,32,45] and three studies addressing aphasia [35,48,51]. In addition, as a secondary outcome, five studies assessed the impact of stimulation on the improvement of functional ability [40,41,42,43,45] and only one study evaluate the effect of cerebellar tDCS on cognitive rehabilitation after stroke [36] (Table 1).

### 3.4. Overview of the Application of Cerebellar NIBS

In this review, we examined fourteen studies focused on the effects of various TMS protocols in cerebellar stroke rehabilitation. These included repetitive TMS [29,30,31,32,39,45], intermittent theta burst stimulation (iTBS) [40,41,42,43,44,46,50], and paired associative stimulation (PAS) [34]), along with eight studies investigating tDCS effects [33,35,36,47,48,49,51,52].

The application of cerebellar TMS in cerebral stroke rehabilitation, according to the contemporary literature, has concentrated on gait–balance, spasticity, and dysphagia as the main outcomes. For post-stroke gait and balance dysfunction, four studies applied iTBS, ranging from 900 to 1200 pulses per treatment [40,43,44,50], and two studies applied 1 Hz rTMS [39,46]. All stimulation sites were located in the contralesional cerebellum. In addition, TMS was employed in every study analyzing spasticity outcomes [41,42]. One study utilized a three-pulse string of 50 Hz continuous theta burst stimulation (cTBS) [42], while another employed ten sessions of 600 pulses of iTBS per session [41]. High-frequency TMS was utilized in each study investigating post-stroke dysphagia [29,30,31,32,45]. Among these, four studies used rTMS at an intensity of 5 to 10 Hz, with the stimulation sites targeting the unilateral or bilateral pharyngeal motor representation of the cerebellum [30,31,32,45]. In the other one study, iTBS was applied to the above bilateral site, using a total of 600 pulses per treatment [29]. Furthermore, for exploring upper limb motor function recovery, PAS between the contralesional cerebellum and the ipsilesional motor cortex was utilized [34]. PAS is a classical conditioning test TMS paradigm, capable of inducing spike-time-dependent plasticity changes between two nodes [53].

In addition, all included tDCS studies in this review implemented anodal cerebellar stimulation, with current intensities ranging from 1.5 mA to 2 mA [33,35,36,47,48,49,51,52]. Cerebellar tDCS has been applied in three studies investigating post-stroke gait and balance impairments [36,49,52], in one study on upper limb dexterity [47], and in all three aphasia studies [35,48,51]. In the studies focusing on motor rehabilitation, barring a single study that used two different bilateral ctDCS montages targeting the dentate nucleus and lower limb representation lobules (VIIb-IX) [52], the anodal electrode was placed 3 cm laterally of the inion (limb representations of the cerebellum), and the cathodal electrode was placed on the buccinator muscles [36,47,49]. In studies regarding aphasia, the stimulation site was 4 cm lateral and 1 cm inferior below the inion on the right cerebellum [35,48,51]. This location aligns roughly with the projection of the cerebellar lobule VII into the scalp. The duration of cerebellar NIBS treatments ranged from a minimum of three days to a maximum of four consecutive weeks, five times a week [36,47].

### 3.5. Motor Symptoms

#### 3.5.1. Gait and Balance

Balance is considered an aspect of postural adaptation, and the cerebellar hemispheres play an important part in motor adaptation [54]. The study identified nine research studies on cerebellar NIBS focusing on gait and balance. Among these, six were TMS studies [39,40,43,44,46,50] and three were tDCS studies [36,49,52] (Table 2). Koch et al. found that patients with hemiplegia due to cerebral infarction exhibited significant improvements in gait and reduced fall risk after receiving contralesional cerebellar 5 Hz iTBS in the chronic phase. Enhanced neural activity was also observed in the posterior parietal cortex of the lesioned hemisphere in the cerebellar iTBS group, indicating restructuring of the brain structure and altered brain function [40]. Following the same iTBS protocol, studies by Liao [43] and Xie [44] also reported significant differences in balance and gait improvement in the iTBS group compared to the sham group, as measured using the 10 m walk test (10-MWT) and Berg Balance Scale (BBS), respectively. In addition, a recent study by Im et al. further explored the effect of low frequency (1 Hz) cerebellar rTMS on post-stroke balance impairment. This study revealed that, compared with the sham group, performance on BBS improved significantly in the rTMS group while the 10-MWT score did not show differences between the two groups, indicate that low-frequency cerebellar rTMS affected balance rather than gait function in infarction patients [46]. Cerebellar TMS has also been used to target balance and gait functions in patients with posterior circulation stroke, suggesting a potential novel and feasible strategy to promote motor learning and improve ataxic symptom [39,50].

Cerebellar tDCS protocols were utilized in the other three studies and showed improvements in balance and gait function. Zandvliet et al.’s innovative work explored the effects of ipsi- and contralesional anodal cerebellar tDCS on 15 patients with balance impairments due to supratentorial infarction. Active contralesional stimulation demonstrated enhanced tandem stance performance compared to the sham group [49]. Similarly, Solanki et al. investigated the lobule-specific impacts of cerebellar tDCS on post-stroke gait performance using two distinct bilateral ctDCS montages targeting the dentate nuclei and lower-limb representation lobules (VIIb-IX). Both montages resulted in improved clinical outcomes, with a direct relationship observed between the mean electric field strength in the lobules and changes in the quantitative gait parameters [52]. A recent study further compared the effects of cerebellar and motor cortex stimulation on gait, balance, and risk of falls, concluding that both sites of stimulation yield similar impacts on mobility in stroke patients [36].

#### 3.5.2. Muscle Spasticity

By modulating the corticospinal excitability via cerebellar-dentato-thalamo-cortical pathways, cerebellar NIBS has also emerged as a potential intervention for spasticity [55]. Two randomized controlled trial of cerebellar TMS on post-stroke spasticity were identified in this study [41,42] (Table 3). In detail, the study of Chen et al. examined the effect of cerebellar iTBS on upper limb spasticity in the acute phase of cerebral infarction. The results indicated a significant decrease in the modified Ashworth scale (MAS), the modified Tardieu scale (MTS), and upper limb elastography values in the iTBS group compared to the sham group, suggesting that ipsilesional cerebellar iTBS could enhance the benefits from physical therapy for post-stroke upper limb spasticity [41]. Moreover, Li et al. illustrated that cerebellar continuous TBS produced similar positive effects on muscle spasticity and limb dyskinesia as low-frequency rTMS (LF-TMS) targeting the contralesional cerebral M1 in chronic stroke patients. Additionally, combining cerebral M1 rTMS with cTBS led to superior outcomes, outperforming the effects of each technique used in isolation [42].

#### 3.5.3. Upper Limb Dexterity

Three studies examined the impact of cerebellar NIBS on upper extremity function following stroke [33,34,47] (Table 4). All three studies recruited supratentorial infarct patients in the chronic phase. One study utilized TMS stimulation [34], while the other two employed tDCS stimulation as the intervention method [33,47]. Rosso et al. applied cerebellar M1 PAS in stroke patients whose Fugl–Meyer Assessment-Upper Extremity (FMA-UE) score was <60 [34]. This TMS paradigm involved applying a conditioning stimulus over the contralesional cerebellum and a test stimulus over ipsilesional M1 cortex, known to induce long-term, potentiation/depression-like plastic changes [56]. Hand function was assessed by using the Jebsen Taylor Test (JTT) and grip strength (GS) was evaluated using a digital analyzer. Following the PAS intervention, a notable improvement in hand coordination and dexterity was observed, although there was no significant increase in grip strength. This improvement correlated with increased activation in the primary motor cortex on the same side of the lesion, suggesting that cerebellar–motor PAS positively contributes to post-stroke hand function recovery [34]. Wessel et al. compared sequential multifocal tDCS (stimulation sequence: M1-CB-M1-CB) to mono-focal tDCS (M1-sham-M1-sham) in chronic stroke patients with motor deficits and found that multifocal tDCS enhanced motor performance in the early training phase. Post hoc analyses further revealed that stroke patients with lower baseline motor skills and sustained cortical disinhibition in the chronic phase derived the greatest benefits from the therapy [33]. Gong et al. also discovered a significant positive effect of right cerebellar tDCS on upper limb motor function, as assessed using the FMA-UE [47]. In subgroup analyses of this study, contralesional cerebellar stimulation was found to be more effective than ipsilesional stimulation.

### 3.6. Other Symptoms

#### 3.6.1. Dysphagia

Five studies were analyzed to assess the effectiveness of NIBS targeting the cerebellum in rehabilitating post-stroke dysphagia (PSD) [29,30,31,32,45] (Table 5). All studies involved patients in the acute or subacute stage of stroke, with high-frequency TMS being the intervention used in each study. A groundbreaking study from Zhong et al. highlighted the therapeutic effects of 5 Hz rTMS on the cerebellum in stroke patients with dysphagia. They found that this intervention yielded comparable results to stimulating both the unaffected and affected mylohyoid cortical regions in terms of improving swallowing function. This underscores the potential of cerebellar rTMS as a safe and effective treatment for post-stroke dysphagia [45]. Subsequent studies by Zhong et al. supported the efficacy of bilateral, cerebellar, high-frequency TMS in treating post-stroke dysphagia [32]. Additionally, Rao et al. studied 70 patients with endoscopically confirmed dysphagia and investigated the effects of a bilateral cerebellar iTBS protocol. The study revealed a significant improvement in the real-iTBS group compared to the control, with the level of enhancement almost matching the clinical difference between using a nasogastric tube for feeding versus oral intake, or relying on parenteral fluids for fluid intake [29]. Dong et al. and Dai et al. compared the efficacy of unilateral and bilateral stimulation, finding both to be effective in improving swallowing function in stroke patients [30,31]. Meanwhile, bilateral stimulation increased the excitability of the cerebral swallowing cortex more significantly compared with unilateral cerebellar rTMS.

#### 3.6.2. Aphasia

Given the anatomical and functional connections between the right cerebellar hemisphere and core language regions, cerebellar NIBS has been applied in the rehabilitation of speech and language functions [57]. Three studies were identified to examine the effect of cerebellar tDCS on post-stroke aphasia [35,48,51] (Table 6). Sebastian et al. provided right cerebellar tDCS treatment to 21 post-stroke aphasia patients and observed a significant improvement in naming tasks two months post-intervention, indicating that this method enhances language expression skills [51]. Similarly, Marangolo et al. conducted a study involving stroke patients with non-fluent aphasia and explored the impact of applying cathodal tDCS to the right cerebellum while simultaneously undergoing language training. The results showed that active stimulation led to significantly greater enhancements in a verb generation task compared to the sham group, suggesting the efficacy of cerebellar tDCS in enhancing language abilities involving non-linguistic strategies [48]. However, in a recent study involving 24 patients with chronic post-stroke aphasia, the use of anodal cerebellar tDCS did not show improvements in language processing immediately after treatment or after a 3-month follow-up period [35].

### 3.7. Functional Ability

Functional ability was evaluated in five studies that examined the impact of cerebellar NIBS on post-stroke balance impairment [40,43], muscle spasticity [41,42], and dysphagia [45]. Changes in functional ability were measured using the Barthel Index (BI), the modified BI, and basic ADL, and improvements were observed across all studies compared to pre-cerebellar stimulation treatment. However, in three studies, no significant differences were observed in BI and basic ADL scores between the groups that received cerebellar stimulation and those that underwent sham stimulation [41,43,45]. A possible explanation for these results may be the lack of adequate cerebellar stimulation.

### 3.8. Adverse Effects

A total of eight research studies reported adverse effects (AEs) following NIBS [29,31,34,36,39,45,47,51]. Two of the studies utilized the tDCS Adverse Effects Questionnaire [58] and Wong–Baker FACES Pain Rating Scale [59] to document side effects and assess pain levels during stimulation [36,51]. The other studies did not describe the questionnaire or scale they applied in the methods section. AEs were not reported during or after stimulation in two studies [34,39]. Among the studies that did report adverse effects, common occurrences included mild pain such as tingling and itching sensations under the electrode [31,36,47,51], slight dizziness [29], transient headache [36,45], and skin redness [36]. These effects were typically temporary, dissipating after stimulation ended or within a few hours, without the need for specific treatment. None of the participants withdrew from the studies due to intolerance. Furthermore, three studies indicated that there were no notable differences in the occurrence of AEs between the control and stimulation groups [29,47,51].

## 4. Discussion

This review focused on the effect of cerebellar NIBS on post-stroke motor and non-motor dysfunction. The studies included in the current review have offered a promising perspective for utilizing this rehabilitation approach following stroke. In terms of motor function, improvement is observed in patients suffering from gait and balance disorders, muscle spasticity, and upper limb dyskinesia. In the other function domain, most studies have reported positive effects on dysphagia. Initial investigations on PSA indicated the improvement in naming and retrieval abilities. Only one study explored the effect of cerebellar NIBS on cognitive functions as a second outcome, and they found a negative result. In addition, it is worth noting that the efficacy of stimulation is influenced by various factors such as different protocols, stroke severity and location, and the timing of therapeutic interventions.

In the context of stroke recovery, NIBS has been found to modulate neural excitability, promote synaptic plasticity, and enhance functional recovery [28]. NIBS can directly influence the excitability of neurons in the targeted brain areas by inducing changes in membrane potential and neurotransmitter release. For instance, it can regulate gamma-aminobutyric acidergic interneuron transmission and boost the expression of brain-derived neurotrophic factor, which serves a neuroprotective function for molecules involved in maintaining blood–brain barrier integrity and supports neural circuit reorganization [60,61]. Moreover, NIBS has been shown to facilitate neuroplastic changes in the brain, allowing for the formation of new neural connections and the strengthening of existing synapses [62]. Preclinical investigations have revealed that stimulating the hemisphere opposite the lesion can enhance perilesional neurogenesis and elevate levels of factors critical for subsequent plasticity mechanisms [62]. NIBS can also influence the connectivity between different brain regions, facilitating the integration of neural networks involved in motor and cognitive processes [63,64]. By enhancing functional connectivity, NIBS may prompt the activation of alternative neural pathways to offset deficits triggered by strokes [65]. NIBS has primarily been used on the primary motor cortex of stroke patients, demonstrating increased effectiveness when paired with supplementary motor training rehabilitation [66].

While the primary motor cortex region has long been a key focus for NIBS techniques in stroke rehabilitation, there is growing interest in exploring the potential of the cerebellum as an additional target for neurorehabilitation [27,67,68]. The use of M1 NIBS is based on the interhemispheric inhibition model, which suggests that disrupting inhibition from the unaffected hemisphere can enhance stroke recovery [69]. However, the effectiveness of these treatment protocols in promoting motor recovery post-stroke may be limited [17,70]. This could be due to cortical injuries that occur after stroke disrupting the electrical field in unpredictable ways, potentially impeding the delivery of stimulation to perilesional tissue crucial for optimal recovery [71].

In order to overcome these limitations, exploring alternative stimulation sites like the cerebellum may offer a promising strategy [28,72,73,74]. One comparative meta-analysis showed stimulations between motor cortex and cerebellar are both effective in improving motor function in stroke patients [25]. Besides its remote position relative to the lesion, the compact structure of the cerebellum makes it possible for anatomically small districts to send diffuse connections to the cerebral cortex [75]. Several clinical studies have also indicated the advantage of cerebellar stimulation; they found a significantly greater improvement in walking and motor capacities in stroke patients who received cerebellar stimulation than patients who received cerebral stimulation [43,76].

Supratentorial ischemic stroke leads to a decrease in cerebral blood flow and metabolism in the hemisphere of the cerebellum opposite the affected side, a phenomenon known as crossed cerebellar diaschisis (CCD) [77,78]. CCD relates to either direct or indirect damage to the corticocerebellar tracts and serves as a marker for clinical deterioration [74,79]. Additionally, damage in the cerebello-thalamo-cortical pathway may result in an imbalance in cerebellar brain inhibition (CBI) [80]. This refers to the cerebellar natural tonic inhibition of the cerebral cortex, causing disruptions in motor control and cognitive functions [80]. Both balancing CCD and modulating CBI are identified as neurophysiological mechanisms for implementing cerebellar stimulation in neurorehabilitation [81,82]. As demonstrated previously, neuronal spiking activity and reduced blood flow in the lesion area can be adjusted by using cerebellar NIBS [83]. Moreover, the cerebellum plays a role in both motor and non-motor functions by facilitating the regulation of the internal learning model, which is also acknowledged as one of the mechanisms of cerebellar NIBS in stroke recovery [2,28]. Cerebellar stimulation can also remodel the cortical cortex by enhancing the functional connectivity in cerebellocortical networks and modulating the amplitudes of motor evoked potentials (MEPs) [75,84,85].

Various cerebellar NIBS techniques have been implemented in the current review. The most common ones include tDCS, iTBS, and rTMS. By using scalp electrodes, tDCS applies a subthreshold static electric field to the brain and can modulate cortical excitability through neuronal tissue polarization [86,87]. Anodal stimulation enhances excitability, while cathodal stimulation diminishes it [87]. The current intensity in the included tDCS studies ranged from 1.5 mA to 2 mA. rTMS utilizes electromagnetic induction to induce plastic changes in the brain. Low frequencies (0.2–1 Hz) decrease excitability, while high frequencies (>5 Hz) increase it [88]. In addition to conventional, regular rTMS, TBS involves applying trains of 50 Hz pulses in continuous or intermittent patterns [88]. cTBS leads to temporary excitation suppression, while iTBS enhances excitation [88]. The stimulus intensity was typically set between 80 and 110% of the resting motor threshold (RMT) or 80% of the active motor threshold (AMT). However, the included studies showed that both excitatory iTBS and inhibitory 1-Hz rTMS can improve posture and gait in stroke patients [40,43,44,46,50]. The differences in intervention timing and stimulation mode may account for the varying results between these studies. In addition, there were no severe safety issues in the included studies, with tDCS considered safer than TMS due to its lower likelihood of triggering action potentials at commonly used intensities. Given that NIBS has emerged as a key focus in neurorehabilitation in recent years, safety guidelines have been established for the use of TMS and tDCS [89,90,91]. Future studies in this area should adhere to these established standards.

There is no universally acknowledged criterion for determining the placement of stimulations in the cerebellum. The cerebellar stimulation site chosen in the current study is usually based on previous research, where MRI reconstruction and neuro navigation techniques were utilized [92,93]. For motor function, the stimulation site is typically located in the superior and lateral cerebellum (1–2 cm inferior and 2–3 cm lateral from the inion), which represents the limbs [42]. Additionally, due to the dentate nucleus’s involvement in planning, initiating, and adjusting voluntary movements [94], one tDCS study targeted the dentate nuclei and lower-limb representations as the stimulation site and showed spill-over effects of dentate nucleus stimulation on post-stroke gait dysfunction [52]. In studies on dysphagia, the coil was placed 4.3 cm lateral to and 2.4 cm below the inion, corresponding to the pharyngeal cortical representation area [95]. Research on aphasia also utilizes tDCS stimulation, with the electrode placement following international EEG systems [35,48,51]. Generally, the cathode is positioned on the right side of the cerebellar cortex, situated 1 cm underneath and 4 cm lateral to the inion, correlating to the positioning of the cerebellar lobule VII on the scalp’s surface. Furthermore, there is no broadly accepted norm for the stimulation side [35,48,51]. Most researches focused on motor function choose contralesional cerebellar hemisphere as the stimulation target [39,40,43,44,46]. One included study suggested that stimulating the contralateral cerebellum relative to the lesion is more effective than ipsilateral stimulation in improving motor function in stroke patients with hemiparesis [47]. This may be due to the majority of fibers in the cerebro-cerebellar pathway being crossed, allowing stimulation of the contralateral cerebellum to improve function in the lesion area [96]. For articulation function, research has shown that both unilateral and bilateral cerebellar stimulation can enhance swallowing function in stroke patients [30,31]. However, bilateral stimulation results in a more significant increase in excitability in the cerebral swallowing cortex compared to unilateral stimulation due to the greater amount of stimulation input [30,31].

The cerebellum plays a critical role in the motor network, particularly in controlling support, balance, and locomotion, as well as being essential for locomotor adaptation and learning processes [97,98]. In the current review, cerebellar stimulation has demonstrated a positive impact in enhancing gait and balance, reducing muscle spasticity, and improving limb dexterity in stroke patients. One study further showed a significant increase in ipsilesional hemisphere MEP amplitudes following cerebellar NIBS treatment, indicating a potential enhancement in corticospinal tract excitability and motor cortex function [40]. The cerebello-thalamo-cortical circuit is thought to be crucial in utilizing cerebellar stimulation for balance rehabilitation, with evidence from neuroimaging studies indicating that higher white matter integrity in superior cerebellar peduncle correlates with improved balance function [99,100]. Moreover, the cerebellum may influence spinal neuron activity related to muscle tone regulation by inducing plasticity at the Purkinje cell level, offering a potential mechanism for the effect of cerebellar NIBS on spasticity improvement after stroke [82,101].Additionally, studies have shown the effectiveness of cerebellar NIBS in enhancing fine-motor hand functions following stroke [33,34]. Investigations in healthy subjects have indicated that cerebellar stimulation can impact various motor skill learning aspects, such as pinch forces and rhythmic finger tapping [102,103]. Future research needs to explore whether the same principles apply in different cases, which aspects of skill and timing learning are regulated, and what kind of variables dictate the outcomes of the therapeutic stimulation.

The effect of cerebellar stimulation on PSD has also been explored, with a recent meta-analysis showing that the stimulation location and parameters may not significantly influence this effect [68]. The interruption of the cerebellar-thalamic-cortical loop is considered to be one pathogenesis of PSD [104,105]. Research has shown that cerebellar stimulation can activate the pharyngeal motor cortex, aiding in the enhancement of swallowing function by reversing the suppression of pharyngeal motor evoked potentials (PMEPs) [106]. In addition, rTMS can heighten cerebellar function and enhance its role in fine motor control, resulting in improved precision in the execution of swallowing actions [107]. Additionally, recent findings propose that stimulating the cerebellar vermis could induce temporary inhibition in the bilateral pharyngeal motor cortex area while concurrently enhancing activity in the cortex–neuron pathway, indicating the potential for targeting different regions of the cerebellum for dysphagia rehabilitation [108].

Three primary studies in the current review focused on language functions after stroke, suggesting that the cerebellum might be a potential target for aphasia rehabilitation. The studies targeted the right cerebellar hemisphere as the stimulation location, which is functionally and anatomically connected to the language center in the left hemisphere [109]. Directly enhancing language function, adjusting linguistic brain networks, and facilitating language learning mechanisms are thought to be possible ways to improve aphasia via cerebellar NIBS [110]. Neuroimaging studies in healthy populations have shown that cerebellar stimulation can alter the cerebellar–cerebrum connection in linguistic brain networks [110,111]. A positron emission tomography study revealed increased glucose metabolism in Wernicke’s and Broca’s areas following cerebellar low-frequency rTMS [112]. During cerebellar stimulation, the resting-state functional connectivity between the cerebellum and networks related to reading and speech motor also showed enhancements [110,111]. Furthermore, research by Marangolo et al. demonstrated that cathodal cerebellar stimulation and anodal frontal lobe stimulation exhibit similar patterns of improvement in verbal fluency tasks, supporting the idea that cerebellar tDCS influences frontal language areas by disinhibiting Purkinje cell functions [48]. Additionally, non-linguistic cognitive functions can be implicated in reshaping neural networks that aid aphasia rehabilitation, with the cerebellum playing an important role in cognitive function [113]. It is also possible that cerebellar NIBS could enhance language symptoms by regulating working memory and executive functions [48].

Only one study in the current review evaluated the effect of cerebellar tDCS on cognitive improvement after stroke, and there were no significant differences pre- and post-treatment [36]. Cognitive performance was evaluated using MMSE and MoCA in the study, and the outcome might have been affected by the inherent ceiling effect of these two scales. Cerebellar TMS research in healthy individuals has demonstrated its influence on various cognitive functions, including attention, working memory, episodic memory, and social cognition [112,114,115,116,117]. Cerebellar NIBS has exhibited alterations in functional connectivity between the cerebellum and cerebral areas, and also within cerebral areas [118]. And, a recent investigation revealed that low-frequency TMS stimulation of the cerebellum had an impact on glucose metabolism in cerebral areas associated with cognitive and emotional processes, such as the medial frontal and cingulate gyri [112]. Vascular cognitive impairment is widespread, with an occurrence rate between 50% and70% [119]. Current treatment strategies including the prevention of vascular risk factors and pharmacological treatments still have limitations [120]. NIBS offers a promising approach for cognitive rehabilitation following stroke.

Our systematic review has updated the information regarding the application of cerebellar NIBS on post-stroke symptoms beyond dyskinesia. We provided a comprehensive summary of the impact of cerebellar NIBS on various neurological deficits following stroke, with a primary focus on exploring the plasticity mechanisms within the corticocerebellar system post-stroke. Several limitations exist in the current review. Firstly, it was difficult to perform a robust quality analysis for each category because of the variability of the evaluation indicators. And, despite the use of the same scales in assessing the outcomes of some functions, like motor and swallowing function, the heterogeneity of the population (differing disease durations and lesion sites), the small clinical sample sizes, and the variety in study protocols may contribute to potential bias if a meta-analysis is employed. Hence, in such cases, providing a qualitative review of the literature may be a more appropriate and objective way to summarizing the results. And, further empirical studies with a larger sample size are needed in the future for potential replications. Nevertheless, the findings from the included studies still provide optimistic prospects for the application of cerebellar NIBS in stroke rehabilitation. Additionally, due to the absence of follow-up studies, the long-term impact of this therapy remains unobserved, which might also account for the non-significant differences observed in the functional ability between the stimulation and sham groups. So, longitudinal studies are necessary to investigate the long-term effects of sustained cerebellar NIBS treatment. Moreover, a mere handful of studies included changes in cortical activity as an outcome [39]. Consequently, future studies employing neuroimaging and electroencephalogram methods are warranted to shed light on the neurophysiological mechanisms underlying cerebellar NIBS. Lastly, factors such as lesion size and location, white matter tract structural integrity, stroke onset duration, and the severity of neurological deficits have been confirmed to influence the stimulation response [2]. Therefore, future research should aim to develop individualized optimizations based on the above variables in order to achieve better therapeutic effects.

## 5. Conclusions

In this systematic review, we compiled cerebellar NIBS studies conducted on stroke patients over the past decade, highlighting the significant role of cerebellar stimulation in neurorehabilitation. We indicated methodological considerations such as the location and type of stimulation that researchers need to consider when designing a cerebellar NIBS protocol, emphasizing the aspects most pertinent to cerebellar stimulation. The potential mechanisms, including the plasticity characteristics and the vast interconnections with cortical areas, were also discussed, suggesting the cerebellum as a key area to target in stroke rehabilitation. Future studies need to develop more precise and customized applications for cerebellar NIBS.

## Figures and Tables

**Table 1:** Focus on points of interest of the included articles.

Number	Author, Year	Focus							
		Muscle Spasticity	Gait	Balance	Hand Function	Dysphagia	Aphasia	Cognitive Function	Functional Ability
1	Kim, 2014 [39]		√	√					
2	Marangolo, 2017 [48]						√		
3	Zandvliet, 2018 [49]		√	√					
4	Koch, 2019 [40]		√	√					√
5	Liao, 2020 [43]		√	√					√
6	Sebastian, 2020 [51]						√		
7	Bonnì, 2020 [50]	√							
8	Zhong, 2021 [45]					√			√
9	Xie, 2021 [44]		√						
10	Li, 2021 [42]	√		√					√
11	Chen, 2021 [41]	√							√
12	Solanki, 2021 [52]		√	√					
13	Rao, 2022 [29]					√			
14	Rosso, 2022 [34]		√	√	√				
15	Im, 2022 [46]		√	√					
16	Dong, 2022 [30]					√			
17	DeMarco, 2022 [35]						√		
18	Qurat-ul-ain, 2023 [36]		√	√				√	
19	Gong, 2023 [47]	√							
20	Wessel, 2023 [33]	√			√				
21	Dai, 2023 [31]					√			
22	Zhong, 2023 [32]					√			

**Table 2:** Clinical studies concerning cerebellar NIBS applications in post-stroke gait and balance dysfunction.

Number	Studies, Year	Sample Size	Stimulation Type and Parameters	Stimulation Location	Outcome Assessments	Main Findings
1	Kim, 2014 [39]	CS: n = 22; SS: n = 10	rTMS, 100% of RMT, 1 Hz, 900 pulses, 5 sessions for 5 consecutive days.	At 2 cm lateral, 2 cm below inion in cerebellum ipsilateral to the ataxia side.	BBS; 10 MWT	Percentage changes after therapy for time and steps in the 10 MWT and BBS between CS vs. SS group: −16.7 ± 35.1% vs. −8.4 ± 72.5%, −8.5 ± 23.0% vs. −0.3 ± 28.4%, and 46.4 ± 100.2% vs. 36.6 ± 71.6%.
2	Zandvliet, 2018 [49]	CS: n = 30; SS: n = 15	tDCS, anodal stimulation, 1.5 mA, 3 sessions for 20 min of 5 consecutive days.	Anodal electrode at 3 cm lateral of the inion.	BBS; TUG; EmNSA-LE; FES; FMA-LE; MI-LE; VAS; CoP	A decrease in CoP composite score in the tandem position was found after CS: β = −0.25, *p* = 0.03.
3	Koch, 2019 [40]	CS: n = 18; SS: n = 18	iTBS, 80% of AMT, 5 Hz, 600 pulses, 2 sessions for 3 consecutive weeks.	Contralesional cerebellar hemisphere.	BBS; BI; FMA; locomotion assessment	The BBS score and step width in gait analysis were compared pre- and post-CS: 34.5 ± 3.4 vs. 43.4 ± 2.6 (*p* < 0.05) and 16.8 ± 4.8 vs. 14.3 ± 6.2 (*p* < 0.05). There were no significant differences observed in FMA and BI between pre- and post-CS.
4	Bonnì, 2020 [50]	CS: n = 8; SS: n = 8	iTBS, 80% of AMT, 600 pulses, 2 sessions, at least once a week.	At 3 cm lateral, 1 cm below inion.	Visuo-motor adaptation task	The rate of error reduction in visuo-motor learning and re-adaptation task between CS vs. SS group: 1.14 ± 0.33 vs. 0.31 ± 0.12 (*p* = 0.03) and 1.33 ± 0.31 vs. 0.47 ± 0.16 (*p* = 0.04). No difference was found in the de-adaptation phase between the two groups.
5	Liao, 2020 [43]	CS: n = 15; SS: n = 15	iTBS, 80% of AMT, 600 pulses, 1 session for 10 days.	At 3 cm lateral, 1 cm below inion in contralesional cerebellum.	BBS; TIS; FMA-LE; BI	All clinical scores significantly increased after CS. The scores in BBS (*p* < 0.001) and TIS (*p* < 0.05) improved more in the CS group than in the SS group.
6	Xie, 2021 [44]	CS: n = 18; SS: n = 18	iTBS, 80% of AMT, 5 Hz, 600 pulses, 2 session for 10 consecutive days.	At 3 cm lateral, 1 cm below inion in contralesional cerebellum.	FMA-LE; 10 MWT; TUG; FAC	Walking performance significantly improved over time and between groups. FMA-LE scores marginally progressed in both groups with no differences observed between groups or across time.
7	Solanki, 2021 [52]	CS: n = 10; SS: n = 10	tDCS, 2 bilateral montages that applied 2 mA for 15 min.	Dentate nuclei, lower-limb representations (lobules VIIb-IX).	10 MWT; TUG; BBS	Overground gait performance improved after CS and is correlated with lobular electric field strength (r = 0.66).
8	Im, 2022 [46]	CS: n = 16; SS: n = 16	rTMS, 90% of RMT, 1 Hz, 900 pulses, 1 session, 5 times per week for 2 weeks.	At 2 cm lateral, 2 cm below inion in the contralesional cerebellum.	BBS; TUG; 10 MWT; ABC	All clinical scores significantly increased after CS therapy. BBS and ABC scores (*p* < 0.05) showed greater improvement in the CS group compared to the SS group. There were no significant differences in the changes observed in the 10 mWT and TUG between the two groups.
9	Qurat-ul-ain, 2023 [36]	CS: n = 22; MS: n = 22; SS: n = 22	tDCS, anodal stimulation, 2 mA, 3 sessions for 20 min of 3 days.	At 1–2 cm below inion occipital protuberance.	BBS; TUG; 6 MWT; 25 FWT; JHFRA; BESTest; MMSE; MoCA	The performance of BBS, TUG, and BESTest significantly improved for both the MS and CS group, demonstrating similar effects. However, neither stimulation induced notable improvements in MMSE and MoCA.

NIBS: noninvasive brain stimulation; CS: cerebellar stimulation; SS: sham stimulation; MS: primary motor cortex stimulation; rTMS: repetitive transcranial magnetic stimulation; RMT: resting motor threshold; BBS: Berg Balance Scale; 10 MWT: 10 m walk test; tDCS: transcranial direct current stimulation; TUG: Timed Up and Go; EmNSA-LE: Erasmus modification of the Nottingham Sensory Assessment Lower Extremity; FES: Fall Efficacy Scale; FMA-LE: Fugl–Meyer assessment lower extremity; MI-LE: Motricity Index of the Lower Extremity; VAS: visual analog scale; CoP: center of pressure; iTBS: intermittent θ-burst stimulation; BI: Barthel Index; TIS: trunk impairment scale; FAC: functional ambulation category scale; ABC: Activity-specific Balance Confidence scale; 6 MWT: Six-Minute Walk Test; 25 FWT: 25-Feet Walk Test; JHFRA: Johns Hopkins Fall Risk Assessment Tool; BESTest: Balance Evaluation Systems Test; MMSE: Mini-Mental State Examination; MoCA: Montreal Cognitive Assessment; AMT: active motor threshold.

**Table 3:** Clinical studies concerning cerebellar NIBS applications in spasticity following stroke.

Number	Studies, Year	Sample Size	Stimulation Type and Parameters	Stimulation Location	Outcome Assessments	Main Findings
1	Chen, 2021 [41]	CS: n = 16; SS: n = 16	iTBS, 80% AMT, 600 pulses, 10 sessions, 5 times a week for 2 weeks.	At 3 cm lateral, 1 cm below inion.	MAS, MTS, SWV, BI	Compared with SS group, CS group had better performance in MAS (*p* < 0.01), MTS (*p* < 0.001) and SWV (*p* < 0.05).
2	Li, 2021 [42]	CS: n = 30; MS: n = 30; CS + MS: n = 30	cTBS, 80% of AMT, 3-pulse bursts at 50 Hz cTBS, 20 days.	At 3 cm lateral, 1 cm below inion in right cerebellum.	MAS, FMA, MBI	Improvements were shown in MAS, FMA, and MBI after therapy in all three groups. CS+MS group showed a lower MAS score and higher FMA and MBI scores than the MS group and CS group.

NIBS: noninvasive brain stimulation; CS: cerebellar stimulation; SS: sham stimulation; MS: primary motor cortex stimulation; iTBS: intermittent θ-burst stimulation; AMT: active motor threshold; MAS: the modified Ashworth scale; MTS: the modified Tardieu scale; SWV: shear wave velocity; BI: Barthel Index; cTBS: continuous theta burst stimulation; FMA: the Fugl–Meyer Assessment; MBI: Modified Barthel Index.

**Table 4:** Clinical studies investigating the impact of cerebellar NIBS on upper extremity function after stroke.

Number	Studies, Year	Sample Size	Stimulation Type and Parameters	Stimulation Location	Outcome Assessments	Main Findings
1	Rosso, 2022 [34]	CS: n = 14; SS: n = 13	PAS, 50% of the maximal stimulator output, 120 pairs of 0.2 Hz stimuli, 5 sessions.	At 3 cm lateral to the inion in the contralesional cerebellum.	JTT; GS	Significant effect of group × time interaction in JTT (*p* = 0.04) was shown, but not in GS (*p* = 0.54). Improved JTT linked to increased ipsilesional motor cortex activation (*p* = 0.04).
2	Gong, 2023 [47]	CS: n = 37; SS: n = 35	tDCS, anodal stimulation, 2 mA for 20 min, 1 session, 5 days a week for 4 weeks.	At 3 cm right lateral to the inion in the right cerebellum.	FMA-UE	Post-stimulation changes in FMA-UE between the CS and SS groups at day 1 and 60 post-therapy: 10.7 ± 1.4 vs. 5.8 ± 1.3 (*p* = 0.01) and 18.9 ± 2.1 vs. 12.7 ± 2.1 (*p* = 0.04). The stimulation effect was more pronounced in patients with right hemiplegia (*p* = 0.03). Different age groups did not show difference between groups (*p* = 0.66).
3	Wessel, 2023 [33]	CS: n = 11; MS: n = 11	tDCS, anodal stimulation; 2 mA for 20 min; fade-in/out interval, 8 s	CS: 3 cm lateral to the inion in the cerebellum ipsilateral to the affected hand; MS: contralateral to the affected hand.	SGFMT	Sequential multifocal tDCS of M1 and CB improved motor performance in a hand-based, sequential motor task in chronic stroke survivors.

NIBS: noninvasive brain stimulation; CS: cerebellar stimulation; SS: sham stimulation; MS: primary motor cortex stimulation; PAS: paired associative stimulation; tDCS: transcranial direct current stimulation; M1: primary motor cortex; JTT: Jebsen–Taylor hand function test; GS: grip strength; FMA-UE: Fugl–Meyer Assessment-Upper Extremity; SGFMT: sequential grip force modulation task.

**Table 5:** Clinical studies concerning cerebellar NIBS applications in post-stroke dysphagia.

Number	Studies, Year	Sample Size	Stimulation Type and Parameters	Stimulation Location	Outcome Assessments	Main Findings
1	Zhong, 2021 [45]	CS: n = 34; Unaffected MS: n = 38; Affected MS: n = 36; SS: n = 35	rTMS, 110% RMT, 5 Hz, 1800 pulses, 1 session for 10 consecutive days.	At 4.3 cm lateral, 2.4 cm below inion.	FEDSS; SSA; PAS; GUSS	Significant time and intervention interaction effects were found for the FEDSS, PAS, SSA, and GUSS scores in all groups (*p* < 0.05). Compared with the SS group, improvements in the above scale scores were shown in the CS, unaffected, and affected MS group (*p* < 0.05).
2	Dong, 2022 [30]	Unilateral CS: n = 12; Bilateral CS: n = 12; SS: n = 12	rTMS, 80% RMT, 10 Hz, 250 pulses, 1 session, 5 days a week for 2 weeks.	At 3 cm lateral, 1 cm below inion.	PAS; FDS	Scores for PAS and FDS improved after therapy in both unilateral and bilateral CS groups (*p* < 0.05), while no significant change was observed in the SS group. Compared to the SS group, improvements in the aforementioned scales also showed in both unilateral and bilateral CS groups (*p* < 0.05), with no significant difference between the two CS groups.
3	Rao, 2022 [29]	Bilateral CS: n = 33; SS: n = 33	iTBS, 100% RMT, 3 pulses of 50 Hz stimulation and repeated at 5 Hz, 600 pulses, 1 session, 5 times a week for 2 weeks.	At 4.3 cm lateral, 2.4 cm below inion.	FEDSS; PAS; SSA; FOIS	Significant time and group interaction effects showed in FEDSS, PAS, SSA, and FOIS score (*p* < 0.001). Compared to the SS group, the scores in the above scales at 2 weeks and 4 weeks significantly improved in the CS group (*p* < 0.05). All scores were significantly improved over time in both CS and SS groups (*p* < 0.001).
4	Dai, 2023 [31]	Bilateral CS: n = 14; Unilateral CS: n = 14; SS: n = 14	rTMS, 90% RMT, 5 trains of 50, 10 Hz stimuli at an intratrain interval of 10 s, 1 session, 5 times a week for 2 weeks.	At 3 cm lateral, 1 cm below inion.	FOIS; DOSS; PAS	Significant time and intervention interaction effects were observed for the FOIS score (*p* = 0.02). Post therapy, the changes in the FOIS scores were significantly higher in the bilateral CS group compared to the SS group (*p* < 0.05). Similarly, greater changes in the DOSS and PAS scores were observed in both the unilateral and bilateral CS groups (*p* < 0.05). Bilateral corticobulbar tract excitability partly increased in the two CS groups, although no significant difference was observed compared to the SS group.
5	Zhong, 2023 [32]	CS: n = 41; SS: n = 43	rTMS, 80% RMT, 10 Hz, 250 pulses, 5 days a week for 2 weeks.	At 2–4 cm anterior, 4–6 cm lateral to the cranial apex.	FEDSS; PAS	The interaction between time and intervention had a significant effect on PAS (*p* < 0.001) and FEDSS (*p* < 0.001). Compared to the SS group, the CS group significantly improved in PAS (*p* = 0.007) and FEDSS (*p* = 0.002).

NIBS: noninvasive brain stimulation; CS: cerebellar stimulation; SS: sham stimulation; MS: primary motor cortex stimulation; rTMS: repetitive transcranial magnetic stimulation; iTBS: intermittent theta burst stimulation; RMT: resting motor threshold; FEDSS: Fiberoptic Endoscopic Dysphagia Severity Scale; SSA: Standardized Bedside Swallowing Assessment; PAS: Penetration/Aspiration Scale; GUSS: Gugging Swallowing Screen; FOIS: Functional Oral Intake Scale; DOSS: Dysphagia Outcome and Severity Scale; FDS: functional dysphagia scale.

**Table 6:** Clinical studies concerning cerebellar NIBS applications in post-stroke aphasia.

Number	Studies, Year	Sample Size	Stimulation Type and Parameters	Stimulation Location	Outcome Assessments	Main Findings
1	Marangolo, 2017 [48]	CS: n = 12; SS: n = 12	tDCS, anodal stimulation, 2 mA for 20 min, 5 consecutive daily sessions over 4 weeks.	At 4 cm lateral, 1 cm inferior below inion in right cerebellum.	Verb generation and naming task	Significant effects of condition (*p* < 0.01) and time (*p* < 0.001) were observed. The percentage of correct responses increased after treatment in both groups (*p* < 0.01); only the CS group improved in the verb generation task (*p* < 0.001)
2	DeMarco, 2022 [35]	CS: n = 10; SS: n = 14	tDCS, anodal stimulation, 2 mA for 20 min, 5 consecutive days	At 4 cm lateral, 1 cm inferior below inion in right cerebellum.	WAB–R; PS-PDT; PNT; category and letter fluency tasks; CSC; verb generation and naming tasks; motor speech production task.	Cerebellar tDCS did not significantly enhance language processing, measured either immediately following treatment or at the 3-month follow-up.
3	Sebastian, 2020 [51]	CS: n = 21; SS: n = 21	tDCS, anodal stimulation, 2 mA for 20 min, 15 sessions (3–5 sessions per week)	At 4 cm lateral, 1 cm inferior below inion in right cerebellum.	Naming 80 test; PNT.	A significant order × treatment interaction was observed immediately post-treatment (*p* = 0.004) in the Naming 80 test. In PNT, the change in naming accuracy between the CS and SS groups was 9.57 (*p* = 0.016) immediately post-treatment and 10.22 (*p* = 0.012) at 2 months post-treatment.

NIBS: noninvasive brain stimulation; CS: cerebellar stimulation; SS: sham stimulation; tDCS: transcranial direct current stimulation; WAB–R: Western Aphasia Battery–Revised; PS-PDT: picnic scene picture description task; CSC: Cloze Sentence Completion; PNT: Philadelphia Naming Test.

## Data Availability

The processing data are available in the Github: https://github.com/mintant/Systematic-Review/blame/main/Full_article.xlsx (accessed on 2 June 2024).

## Supplementary Materials

The following supporting information can be downloaded at: https://www.mdpi.com/article/10.3390/biomedicines12061348/s1, Table S1: Search strategies; Table S2: Risk of bias judgement.
