# Supplementary material for: Effects of Cerebellar Non-Invasive Stimulation on Neurorehabilitation in Stroke Patients: An Updated Systematic Review
Source: Biomedicines. 2024 Jun 18;12(6):1348. doi: 10.3390/biomedicines12061348 (PMC11201496; doi:10.3390/biomedicines12061348)
Supplement: Supplementary file 1 [file biomedicines-12-01348-s001.zip › biomedicines-2984955-supplementary.pdf]

Table S1. Search Strategies

Search included: PUBMED, EMBASE and WEB OF SCIENCE: search date was from the inception through November 1st 2023

1) Pubmed search strategy

1. "stroke"[MeSH Terms]
2. "brain ischemia"[Title/Abstract]
3. "brain infarction"[Title/Abstract]
4. "cerebral infarction"[Title/Abstract]
5. "intracerebral hemorrhage"[Title/Abstract]
6. "intracranial hemorrhage"[Title/Abstract]
7. 1 OR 2 OR 3 OR 4 OR 5 OR 6
8. "stimulat*"[Title/Abstract]
9. "neurostimulation"[Title/Abstract]
10. "neuromodulat*"[Title/Abstract]
11. "Transcranial Direct Current Stimulation"[Mesh]
12. "Transcranial Magnetic Stimulation"[Mesh]
13. 8 OR 9 OR 10 OR 11 OR 12
14. "cerebell* "[Title/Abstract]
15. 7 AND 13 AND 14

2) Embase search strategy

1. 'stroke'/exp
2. brain Ischemia:ab,ti
3. brain infarction:ab,ti
4. cerebral infarction:ab,ti
5. intracerebral hemorrhage:ab,ti
6. intracranial hemorrhage:ab,ti
7. 1 OR 2 OR 3 OR 4 OR 5 OR 6
8. 'non invasive brain stimulation'/exp
9. 'transcranial direct current stimulation'/exp
10. 'transcranial magnetic stimulation'/exp
11. neuromodulat*:ab,ti
12. stimulat*:ab,ti
13. neurostimulat*:ab,ti

14. 8 OR 9 OR 10 OR 11 OR 12 OR 13
15. cerebell*:ab,ti
16. 7 AND 14 AND 15

3) Web of science search strategy

1. “stroke”
2. “brain Ischemia”
3. “brain infarction”
4. “cerebral infarction”
5. “intracerebral hemorrhage”
6. “intracranial hemorrhage”
7. TS=(1 OR 2 OR 3 OR 4 OR 5 OR 6)
8. “non invasive brain stimulation”
9. “transcranial direct current stimulation”
10. “transcranial magnetic stimulation”
11. “neuromodulat*”
12. “stimulat*”
13. “neurostimulat*”
14. TS=(8 OR 9 OR 10 OR 11 OR 12 OR 13)
15. TS=(cerebell*)
16. 7 AND 14 AND 15

Table S2. Risk of Bias Judgement

Kim et al., 2014

Bias	Authors' Judgement	Justification
Random sequence generation (selection bias)	Low risk	A randomization sequence was generated by a computer.
Allocation concealment (selection bias)	Low risk	Allocation was concealed by opaque envelopes.
Blinding of participants and personnel (performance bias)	Low risk	Patients were blinded to group allocation.
Blinding of outcome assessment (detection bias)	Unclear risk	Whether or not assessor was blinded is not stated.
Incomplete outcome data (attrition bias)	Low risk	All participants completed the study.
Selective reporting (reporting bias)	Low risk	The statistical analysis is consistent with the protocol.
Other bias	Unclear risk	Intention-to-treat used with participants who completed baseline assessments.

Marangolo et al., 2017

Bias	Authors' Judgement	Justification
Random sequence generation (selection bias)	Unclear risk	Randomization method not described.
Allocation concealment (selection bias)	Unclear risk	Concealment of allocation not described.
Blinding of participants and personnel (performance bias)	Low risk	The patients were blinded regarding the stimulation condition.
Blinding of outcome assessment (detection bias)	Low risk	The experimenter and the patient were blinded regarding the stimulation condition.
Incomplete outcome data (attrition bias)	Low risk	All participants completed the study.
Selective reporting (reporting bias)	Low risk	The statistical analysis is consistent with the protocol.

Other bias	Unclear risk	It's a crossover study. Each participant received cerebellar tDCS in four experimental conditions: (1) right cathodal and (2) sham stimulation during a verb generation task and (3) right cathodal and (4) sham stimulation during a verb naming task. Each experimental condition was run in five consecutive daily sessions over 4 weeks.

Zandvliet et al., 2018

Bias	Authors' Judgement	Justification
Random sequence generation (selection bias)	Unclear risk	Randomization method not described.
Allocation concealment (selection bias)	Unclear risk	Concealment of allocation not described
Blinding of participants and personnel (performance bias)	Low risk	The participants were blinded.
Blinding of outcome assessment (detection bias)	Unclear risk	Whether or not assessor was blinded is not stated
Incomplete outcome data (attrition bias)	Low risk	All participants completed the study.
Selective reporting (reporting bias)	Low risk	The statistical analysis is consistent with the protocol.
Other bias	Unclear risk	It's a crossover study. No significant differences at baseline for demographic measures. All participants in this trial follow the same protocol.

Koch et al., 2019

Bias	Authors' Judgement	Justification
Random sequence generation (selection bias)	Low risk	The randomization algorithm used the minimal sufficient balancing method.
Allocation concealment (selection bias)	Unclear risk	Concealment of allocation not described.
Blinding of participants and personnel (performance bias)	Low risk	Participants and personnel were blind to group assignment.

Blinding of outcome assessment (detection bias)	Low risk	Clinical evaluation was performed by a clinician or by a neurophysiologist who was blinded to the experimental condition of the patient.
Incomplete outcome data (attrition bias)	Unclear risk	Dropouts <10% overall (5.6% in treatment, 5.6% in control).
Selective reporting (reporting bias)	Low risk	The statistical analysis is consistent with the protocol.
Other bias	Unclear risk	Participants instructed to continue with physiotherapy in promoting gait and balance recovery.

Liao et al., 2020

Bias	Authors' Judgement	Justification
Random sequence generation (selection bias)	Low risk	All subjects were randomly assigned according to the random number table.
Allocation concealment (selection bias)	Unclear risk	Concealment of allocation not described.
Blinding of participants and personnel (performance bias)	Low risk	The participants were blinded.
Blinding of outcome assessment (detection bias)	Low risk	The assessors were blinded.
Incomplete outcome data (attrition bias)	High risk	Dropouts > 10% (13.3% in treatment group, 20% in control group).
Selective reporting (reporting bias)	Low risk	The statistical analysis is consistent with the protocol.
Other bias	Unclear risk	All participants in this trial underwent the standard physical therapy program.

Sebastian et al., 2020

Bias	Authors' Judgement	Justification
Random sequence generation (selection bias)	Unclear risk	Participants randomized in blocks.
Allocation concealment (selection bias)	Unclear risk	Concealment of allocation not described

Blinding of participants and personnel (performance bias)	Low risk	The participants were blinded.
Blinding of outcome assessment (detection bias)	Unclear risk	Whether or not assessor was blinded is not stated.
Incomplete outcome data (attrition bias)	High risk	Dropout rate is 12.5%
Selective reporting (reporting bias)	Low risk	The statistical analysis is consistent with the protocol.
Other bias	Unclear risk	Seven participants in this study were taking selective serotonin reuptake inhibitors. The individual variability observed in this study could be related to the medications the participants were taking.

Bonnì et al., 2020

Bias	Authors' Judgement	Justification
Random sequence generation (selection bias)	Unclear risk	Randomization method not described
Allocation concealment (selection bias)	Unclear risk	Concealment of allocation not described
Blinding of participants and personnel (performance bias)	Unclear risk	Not described.
Blinding of outcome assessment (detection bias)	Unclear risk	Not described.
Incomplete outcome data (attrition bias)	Low risk	All participants completed the study.
Selective reporting (reporting bias)	Low risk	The statistical analysis is consistent with the protocol.
Other bias	Unclear risk	It might be considered as a pilot study due to the small sample size.

Zhong et al., 2021

Bias	Authors' Judgement	Justification

Random sequence generation (selection bias)	Low risk	Patients were randomly divided into three groups by the random number table method.
Allocation concealment (selection bias)	Low risk	A sealed opaque envelope was opened at patient enrollment to determine the groups.
Blinding of participants and personnel (performance bias)	High risk	Patients were not blinded to the group assignment.
Blinding of outcome assessment (detection bias)	Low risk	This is an observer-blind trail.
Incomplete outcome data (attrition bias)	Unclear risk	Dropouts <10% (1.4% in treatment group; 0 in control group).
Selective reporting (reporting bias)	Low risk	The statistical analysis is consistent with the protocol.
Other bias	Low risk	These groups of patients received the same amount of traditional dysphagia treatment for 30min daily after the intervention, such as thermal tactile stimulation, vocal cord exercises, Shaker exercises, Masako maneuvers, oropharyngeal muscle strengthening exercises, and tongue retraction exercises.

Xie et al., 2021

Bias	Authors' Judgement	Justification
Random sequence generation (selection bias)	Low risk	Participants were randomly assigned by a computer-generated, blockwise random sequence to either the intervention group or the control group with a 1:1 allocation ratio.
Allocation concealment (selection bias)	Low risk	The randomization identification number and treatment allocation code were kept in sealed opaque envelopes.
Blinding of participants and personnel (performance bias)	Low risk	Participants and physical therapists were unaware of the group assignment.
Blinding of outcome assessment (detection bias)	Low risk	The study assessor was unaware of the group assignment.
Incomplete outcome data (attrition bias)	Unclear risk	Dropouts <10% (5.6% in both treatment and control group).
Selective reporting (reporting bias)	Low risk	The statistical analysis is consistent with the protocol.

Other bias	Low risk	iTBS and conventional physical therapy were conducted and supervised by well-trained and qualified physical therapist.

Li et al., 2021

Bias	Authors' Judgement	Justification
Random sequence generation (selection bias)	Unclear risk	Randomization method not described.
Allocation concealment (selection bias)	Unclear risk	Concealment of allocation not described
Blinding of participants and personnel (performance bias)	Unclear risk	Not described.
Blinding of outcome assessment (detection bias)	Unclear risk	Not described.
Incomplete outcome data (attrition bias)	Low risk	No dropouts reported
Selective reporting (reporting bias)	Low risk	The statistical analysis is consistent with the protocol.
Other bias	Unclear risk	It might be considered as a pilot study due to the small sample size.

Chen et al., 2021

Bias	Authors' Judgement	Justification
Random sequence generation (selection bias)	Low risk	The randomization sequences were generated based on the table of random digits.
Allocation concealment (selection bias)	Low risk	Allocation was concealed by opaque numbered envelopes, which were opened in numerical order by a neutral non-involved researcher.
Blinding of participants and personnel (performance bias)	Low risk	Participants and personnel were blind to group assignment.
Blinding of outcome assessment (detection bias)	Low risk	Each evaluation was performed by a clinician or by a physical therapist who was blinded to the experimental condition of the patient.
Incomplete outcome data (attrition bias)	Unclear risk	Dropouts < 10% (12.5% in control group, 6.3% in treatment group).

Selective reporting (reporting bias)	Low risk	The statistical analysis is consistent with the protocol.
Other bias	Low risk	Conventional physical therapy was performed for both groups.

Solanki, et al., 2021

Bias	Authors' Judgement	Justification
Random sequence generation (selection bias)	Unclear risk	Randomization method not described.
Allocation concealment (selection bias)	Unclear risk	Concealment of allocation not described.
Blinding of participants and personnel (performance bias)	Low risk	Patients were blinded to group allocation.
Blinding of outcome assessment (detection bias)	High risk	Assessor not blinded due to purposeful recruitment process.
Incomplete outcome data (attrition bias)	High risk	Dropout rate is 16.7%
Selective reporting (reporting bias)	Low risk	The statistical analysis is consistent with the protocol.
Other bias	High risk	Due to small sample, participants were not similar at baseline despite randomization

Rao et al., 2022

Bias	Authors' Judgement	Justification
Random sequence generation (selection bias)	Low risk	Randomization was performed by a staff member who did not participate in the implementation or evaluation of the trial using a pseudorandom number generated by a computer.
Allocation concealment (selection bias)	Low risk	Opaque, sealed envelopes were used to mask the randomization tables and they contained a paper labeled with a random identification number and the assignment code of the participant.
Blinding of participants and personnel (performance bias)	Low risk	The participants were masked to the treatment condition.

Blinding of outcome assessment (detection bias)	Low risk	The outcome evaluators were masked to the treatment condition.
Incomplete outcome data (attrition bias)	Unclear risk	Dropouts <10% (5.7% in treatment group; 11.4% in control group).
Selective reporting (reporting bias)	Low risk	The statistical analysis is consistent with the protocol.
Other bias	Unclear risk	No traditional swallowing function training was described in this article.

Rosso et al., 2022

Bias	Authors' Judgement	Justification
Random sequence generation (selection bias)	Low risk	A computer-generated randomization was achieved using a secure website (http://randoweb.aphp. fr), which is based on an algorithm that used a minimal sufficient balancing method to prevent imbalances in the three predefined following variables: age, initial Fugl-Meyer score, and side of the lesion.
Allocation concealment (selection bias)	Unclear risk	Concealment of allocation not described.
Blinding of participants and personnel (performance bias)	Low risk	Patients were also blinded to the treatment allocation.
Blinding of outcome assessment (detection bias)	Low risk	Two other blinded investigators were responsible for clinical, electrophysiological, and neuroimaging assessments.
Incomplete outcome data (attrition bias)	Unclear risk	Dropouts <10% overall (6.7% in control group).
Selective reporting (reporting bias)	Low risk	The statistical analysis is consistent with the protocol.
Other bias	Unclear risk	No significant differences at baseline for demographic measures; Physical therapy was performed for both groups.

Im, et al., 2021

Bias	Authors' Judgement	Justification
Random sequence generation (selection bias)	Unclear risk	Randomization method not stated.

Allocation concealment (selection bias)	Unclear risk	Concealment of allocation not described.
Blinding of participants and personnel (performance bias)	Low risk	Patients were blinded to group allocation.
Blinding of outcome assessment (detection bias)	Low risk	Trained, blinded physiotherapist completed assessments.
Incomplete outcome data (attrition bias)	Unclear risk	Dropouts <10 % overall (3.1% in the rTMS group)
Selective reporting (reporting bias)	Low risk	The statistical analysis is consistent with the protocol.
Other bias	Unclear risk	The duration of cerebral infarction varied significantly among subjects.

Dong et al., 2022

Bias	Authors' Judgement	Justification
Random sequence generation (selection bias)	Unclear risk	Randomization method not described.
Allocation concealment (selection bias)	Unclear risk	Concealment of allocation not described.
Blinding of participants and personnel (performance bias)	Low risk	Neither the patients nor the physicians responsible for the evaluation were aware of the distribution of the treatment options in each group.
Blinding of outcome assessment (detection bias)	Low risk	The FDS and PAS scores were determined by a speech therapist who was not informed about the study and patient grouping according to the VFSS.
Incomplete outcome data (attrition bias)	Unclear risk	Dropouts <10% (4.2% in treatment group; 8.3% in control group).
Selective reporting (reporting bias)	Low risk	The statistical analysis is consistent with the protocol.
Other bias	Unclear risk	Traditional swallowing function training included temperature stimulation, air pulse stimulation, taste stimulation, tongue resistance training, and throat lift training, and the training is about 20 minutes after the daily rTMS treatment.

DeMarco et al., 2022

Bias	Authors' Judgement	Justification

Random sequence generation (selection bias)	High risk	Individuals were not randomized to the treatment group.
Allocation concealment (selection bias)	High risk	Individuals were not randomized to the treatment group.
Blinding of participants and personnel (performance bias)	High risk	Participants knew that they were receiving what kind of stimulation.
Blinding of outcome assessment (detection bias)	Unclear risk	Not described.
Incomplete outcome data (attrition bias)	Low risk	No dropouts reported
Selective reporting (reporting bias)	Low risk	The statistical analysis is consistent with the protocol.
Other bias	Unclear risk	It might be considered as a pilot study due to the small sample size.

Qurat-ul-ain, et al., 2023

Bias	Authors' Judgement	Justification
Random sequence generation (selection bias)	Low risk	Central randomization was conducted using computer-generated randomization service.
Allocation concealment (selection bias)	Low risk	Patient allocation was effectuated by an independent administrator.
Blinding of participants and personnel (performance bias)	Low risk	Participants were blinded to group allocation throughout the duration of the trial.
Blinding of outcome assessment (detection bias)	Low risk	Assessors were blinded to group allocation throughout the duration of the trial.
Incomplete outcome data (attrition bias)	High risk	Dropout rate is 12.8%.
Selective reporting (reporting bias)	Low risk	The statistical analysis is consistent with the protocol.
Other bias	High risk	Majority of the outcome measures had baseline clinical heterogeneity.

Gong, et al., 2023

Bias	Authors' Judgement	Justification

Random sequence generation (selection bias)	Low risk	A blocked stratified randomization procedure was used.
Allocation concealment (selection bias)	Unclear risk	Concealment of allocation not described.
Blinding of participants and personnel (performance bias)	Unclear risk	Not described.
Blinding of outcome assessment (detection bias)	Unclear risk	Not described.
Incomplete outcome data (attrition bias)	Unclear risk	Dropout rate is 5.6%
Selective reporting (reporting bias)	Low risk	The statistical analysis is consistent with the protocol.
Other bias	Unclear risk	It might be considered as a pilot study due to the small sample size.

Wessel, et al., 2023

Bias	Authors' Judgement	Justification
Random sequence generation (selection bias)	Unclear risk	Randomization method not described.
Allocation concealment (selection bias)	Unclear risk	Concealment of allocation not described.
Blinding of participants and personnel (performance bias)	Low risk	Participants were blinded to group allocation throughout the duration of the trial.
Blinding of outcome assessment (detection bias)	Low risk	Assessors were blinded to group allocation throughout the duration of the trial.
Incomplete outcome data (attrition bias)	Unclear risk	Dropout rate is 8.3%
Selective reporting (reporting bias)	Low risk	The statistical analysis is consistent with the protocol.
Other bias	Unclear risk	The sample size is rather small for statistical comparisons.

Dai, et al., 2023

Bias	Authors' Judgement	Justification

Random sequence generation (selection bias)	Low risk	Patients were randomly allocated into three groups using computer-generated permuted block randomization, with a block size of 6.
Allocation concealment (selection bias)	Low risk	Participants were blinded to group allocation throughout the duration of the trial.
Blinding of participants and personnel (performance bias)	Low risk	The patients were blinded regarding the stimulation condition.
Blinding of outcome assessment (detection bias)	High risk	Assessor were not blinded.
Incomplete outcome data (attrition bias)	High risk	Dropout rate is 14.3%
Selective reporting (reporting bias)	Low risk	The statistical analysis is consistent with the protocol.
Other bias	Unclear risk	The sample size was relatively small and heterogeneous, and the findings should be replicated with a larger sample size.

Zhong, et al., 2023

Bias	Authors' Judgement	Justification
Random sequence generation (selection bias)	Low risk	Patients were randomly allocated into two groups using a random number table method.
Allocation concealment (selection bias)	Low risk	Allocation was concealed by opaque envelopes.
Blinding of participants and personnel (performance bias)	Low risk	Participants were blinded to group allocation throughout the duration of the trial.
Blinding of outcome assessment (detection bias)	Low risk	Assessors were blinded to group allocation throughout the duration of the trial.
Incomplete outcome data (attrition bias)	Unclear risk	Dropout rate is 1.2%
Selective reporting (reporting bias)	Low risk	The statistical analysis is consistent with the protocol.
Other bias	Unclear risk	Conventional swallowing rehabilitation training was performed for both groups.
